# What are the main environmental exposures associated with elevated IgE in Cuban infants? A population-based study

**DOI:** 10.1111/tmi.12293

**Published:** 2014-03-28

**Authors:** Hermes Fundora-Hernández, Silvia J Venero-Fernández, Ramón Suárez-Medina, Esperanza de la C Mora-Faife, Gladys García-García, Ileana del Valle-Infante, Liem Gómez-Marrero, Andrea Venn, John Britton, Andrew W Fogarty, HINASIC Study Group

**Affiliations:** 1 Instituto Nacional de Higiene, Epidemiología y MicrobiologíaLa Habana, Cuba; 2 Nottingham Biomedical Research Unit, Division of Epidemiology and Public Health, University of Nottingham, City HospitalNottingham, UK

**Keywords:** IgE, infants, risk factor, Cuba, allergy

## Abstract

**Objective:**

Immunoglobulin E (IgE) plays a key role in allergy disease pathogenesis, but little is known about the environmental factors associated with higher IgE levels in infants. The aim of this study was to determine the risk factors for elevated serum total IgE infants living in Havana.

**Methods:**

Eight hundred and seventy-seven infants provided blood samples. Data on allergic disease symptoms and a wide range of exposures were collected.

**Results:**

The median IgE was 35IU/ml (interquartile range 13–96). The risk of having an IgE level above the median was higher for children who had been breastfed for 4 months or more (adjusted odds ratio (OR) 1.28; 95% confidence interval (CI): 1.02–1.61) and for children who reported cockroaches in their home (OR 1.30; 95% CI: 1.03–1.63). The risk was lower for children whose mother was in paid employment (OR 0.73; 95% CI: 0.54–0.97 compared with those who did not), for children living in homes where gas and electricity were used for cooking (OR 0.45; 95% CI: 0.32–0.62 compared with electricity only) and for children with domestic pets at birth (OR 0.83; 95% CI: 0.70-1.00). There was no association between paracetamol use and serum IgE levels.

**Conclusions:**

Associations between gas fuel use and maternal employment indicate that IgE levels in early life are lower in children who may be living in relative affluence. The discrepancy in the effect of early exposure to pets or cockroaches may reflect differences in these allergens, or be confounded by relative affluence. Further investigation of this cohort will determine how these effects translate into the expression of allergic disease in later life.

**Objectif:**

Les immunoglobulines E (IgE) jouent un rôle clé dans la pathogenèse de la maladie allergique, mais on sait peu sur les facteurs environnementaux associés à des taux plus élevés d'IgE chez les nourrissons. Le but de cette étude était de déterminer les facteurs de risque pour un taux élevé d'IgE sériques totales chez les nourrissons vivant à La Havane.

**Méthodes:**

Des échantillons de sang ont été collectés chez 877 nourrissons. Les données sur les symptômes de la maladie allergique et sur une large gamme d'expositions ont été recueillies.

**Résultats:**

La médiane des IgE était de 35 UI/ml (gamme interquartile: 13 à 96). Le risque d'avoir un taux d'IgE au-dessus de la médiane était plus élevé pour les enfants qui avaient été allaités pendant au moins quatre mois (odds ratio ajusté (OR): 1,28; intervalle de confiance (IC) à 95%: 1,02 à 1,61) et pour les enfants pour qui des cafards ont été signalés dans la maison (OR: 1,30; IC 95%: 1,03 à 1,63). Le risque était plus faible pour les enfants dont la mère possédait un emploi rémunéré (OR: 0,73; IC 95%: 0,54 à 0,97 par rapport à ceux dont les mères n'avaient pas cet emploi, pour les enfants vivant dans des foyers où le gaz et l’électricité étaient utilisés pour la cuisson (OR: 0,45; IC 95%: 0,32 à 0,62 par rapport à l'utilisation de l’électricité seule) et pour les enfants ayant des animaux domestiques à la naissance (OR: 0,83; IC 95%: 0,70 à 1,00). Il n'y avait pas d'association entre les taux d'IgE sériques et l'utilisation de paracétamol.

**Conclusions:**

Les associations avec la consommation du gaz carburant et l'emploi de la mère indiquent que les taux d'IgE en début de vie sont plus faibles chez les enfants vivant dans une relative richesse. La différence dans l'effet de l'exposition précoce aux animaux de compagnie ou à des cafards pourrait refléter des différences dans ces allergènes, ou être confondu par la richesse relative. Une investigation plus approfondie de cette cohorte permettra de déterminer comment ces effets se traduisent dans l'expression de la maladie allergique plus tard dans la vie.

**Objetivo:**

La inmunoglobulina E (IgE) juega un papel clave en la patogénesis de la enfermedad alérgica, pero se conoce poco sobre los factores ambientales asociados con unos niveles altos de IgE en los niños. El objetivo de este estudio era determinar los factores de riesgo de unos niveles elevados de IgE en sueros de niños viviendo en La Habana.

**Métodos:**

Se obtuvieron muestras de sangre de 877 niños. Se recolectaron datos sobre los síntomas de la alergia y un amplio rango de exposiciones.

**Resultados:**

La mediana de IgE era de 35 IU/ml (rango intercuartil 13–96). El riesgo de tener unos niveles altos de IgE, por encima de la mediana, era mayor en niños que habían sido amamantados durante cuatro meses o más (odds ratio (OR) ajustado 1.28; intervalo de confianza (IC) 95%: 1.02–1.61) y en niños para los que se había reportado presencia de cucarachas en sus hogares (OR 1.30; 95% CI: 1.03–1.63). El riesgo era menor para niños cuya madre tenía un empleo pagado (OR 0.73; IC 95%: 0.54–0.97 comparado con aquellos que no la tenían), para niños viviendo en hogares en los que se utilizaba gas y electricidad para cocinar (OR 0.45; IC 95%: 0.32–0.62 comparado con electricidad solamente) y para niños con mascotas domésticas en el momento de nacer (OR 0.83; IC 95%: 0.70–1.00). No existía una asociación entre el uso del paracetamol y los niveles de IgE en suero.

**Conclusiones:**

Las asociaciones con el uso de gas como combustible y el empleo de la madre indican que los niveles de IgE en una etapa temprana de la vida son menores en niños viviendo con un cierto nivel de opulencia. Las discrepancias en el efecto de una exposición temprana a mascotas o cucarachas podría reflejar las diferencias en estos alergenos, aunque la relativa opulencia podría jugar como factor de confusión. Nuevos estudios con esta cohorte podrían determinar cómo estos efectos se traducen en la expresión de la enfermedad alérgica más adelante en la vida.

## Introduction

Allergic disease is increasing in prevalence globally and is most common in the more affluent, urbanised and economically developed countries (Asher *et al*. 2006). However, the aetiological factors responsible for this change remain unknown. Immunoglobulin E (IgE) is known to be an important mediator with regard to both allergic disease (Martinez *et al*. 1995; Rodriguez *et al*. 2001; Busse *et al*. 2011) and parasitic disease (Platts-Mills 2001; Cooper *et al*. 2008), and it is considered that the former may represent an aberrant response from an immune system that has evolved in coexistence with parasitic infections (Gould & Sutton 2008). Although investigators have clearly demonstrated that serum IgE is a risk factor for allergic disease in older children (Sears *et al*. 1991; Sporisk *et al*. 1995; Simpson *et al*. 2005), there are few population-based studies with IgE measurement in children under 2 years (Martinez *et al*. 1995), and hence little is known about the risk factors that modify IgE levels in this age group. Hence, environmental factors that modify serum IgE represent an important area of research as these are potentially modifiable risk factors for allergic diseases, and in particular asthma (Sears *et al*. 1991; Sporisk *et al*. 1995; Simpson *et al*. 2005). The study of risk factors for an elevated IgE in the early years of life is particularly important, representing the cumulative impact of the environment on the maturing immune system. The hypothesis that IgE is important in the pathogenesis of asthma has been recently supported by the development of interventions designed to reduce serum IgE in the treatment of asthma in children (Busse *et al*. 2011).

Cuba has an excellent health care system that delivers infant mortality rates comparable with much richer countries (Cooper *et al*. 2006). The combination of good health infrastructure with limited economic growth in recent decades (as a consequence of the economic embargo on the island imposed by the USA) has resulted in a unique environment where risk factors for disease can be studied. In the recent Phase 3 of the International Study of Asthma and Allergies in Childhood (ISAAC), Cuba had one of the highest prevalences of childhood eczema among the countries studied from Latin America (Sole *et al*. 2010). Allergic disease is considered common in Cuba with estimates of prevalences of 32% for recent asthma symptoms and 20% for allergic rhinitis in young boys living in Habana (Venero Fernandez *et al*. 2009). We used a cross-sectional study design to explore the risk factors for increased IgE in a population-based study of one-year-old infants born in Havana, Cuba.

## Methods

### Study population

All children aged 12–15 months who were living in Havana, Cuba, between March 2010 and March 2011 and who attended one of 17 randomly selected policlinics in four municipalities in Havana, Cuba, were eligible to be selected to participate in the study (Arroyo Naranjo, Cerro, Habana del Este, La Lisa). Recruitment for the study is described in detail elsewhere (Venero-Fernandez *et al*. 2013). The study protocol was approved by the National Institute of Hygiene, Epidemiology and Microbiology, the local Havana Scientific Committee in Cuba and the University of Nottingham Medical School Ethics Committee in the United Kingdom.

### Data collection

The study was designed to identify environmental exposures that may increase the risk of asthma or allergic disease in Cuba. The baseline data collection consisted of an interviewer-administered questionnaire that collated the responses from the parent/carer about prenatal and post-natal exposures of the child, their living environment and the medical history of the family. Specific questions focussed on paracetamol exposure by the mother during pregnancy and also detailed information on exposure to individuals who smoke tobacco in the child's living environment. Data on the height and weight at the time of the interview were also collected using the measuring equipment and weight scales available in the Policlinic. For those infants whose parents gave consent a blood sample was taken. This was frozen and subsequently analysed for serum IgE using the UMELISA assay manufactured by TecnoSuma International (Cuba) (http://www.tecnosumacom/Informacion/htm/UM2007.htm, accessed 16/1/2014; Solis *et al*. 1992) in a standardised manner designed to minimise measurement error. Collecting stool samples from infants in a tropical climate are challenging, and many infants were not able to provide a sample for analysis. Where possible, a stool sample was also taken and analysed for current parasite infection using the direct method with eosin and Lugol solutions following protocols from the Pedro Kouri Institute of Tropical Medicine, Havana, based on the established Kato–Katz method to identify *Endolimax lana, Giardia lamblia* and *Entamoeba histolytica* (Habtamu *et al*. 2011).

### Data analysis

The data were entered into an electronic database, cleaned and checked for errors. All statistical analyses were carried out in Stata v12 (StataCorp, TX, USA) using the survey commands to allow for the clustered survey design. The distribution of serum IgE was not normally distributed even after log transformation. As there is no established universal definition for an increased IgE in this age group, the data were divided into above and below the median value and subsequent analyses assessed the odds of an exposure being associated with an IgE above the median value.

Bivariate analyses of all descriptive factors and exposure variables were initially performed using logistic regression and odds ratios, and associated 95% confidence intervals were computed for each exposure variable adjusting for gender and municipality, which were considered *a priori* confounders. Where substantial co-linearity between exposure variables was observed, e.g. between maternal education and household income, one exposure was selected for the final model to maintain model integrity. Variables that were statistically significant in this analysis (*P* ≤ 0.05) were then entered into a multivariable model, and a step-wise modelling procedure followed to obtain a final model of only statistically significant (*P* ≤ 0.05) variables. No further *post hoc* modelling was performed on the data set to maintain transparency and simplicity.

## Results

**Figure 1:**
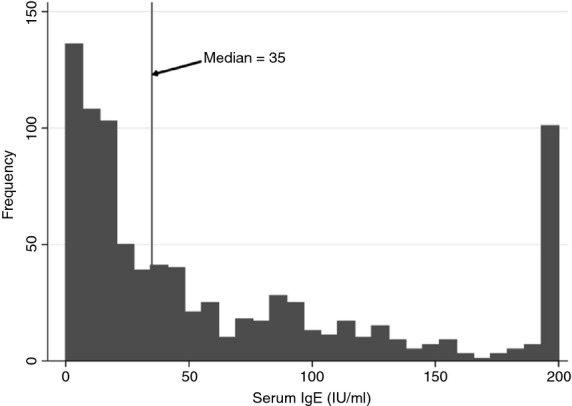
Distribution of serum IgE in study population.

Of the 1956 infants who participated in the original survey, 877 (45%) infants provided blood samples for analysis of serum IgE levels. Children who provided blood were similar to those who did not in terms of age, siblings and mothers' age, but were slightly more likely to be male, have black skin, live in Habana del Este municipality, have a mother who was in paid work and have reported allergic disease symptoms than those who did not (Table1). The median IgE was 35.00 IU/ml (interquartile range 13–96) and the distribution is presented in Figure1.

**Table I:** Characteristics of study participants

Variable	Definition of category	All children: *N* = 1956	Children who did not give blood *N* = 1079	Children who gave blood *N* = 877	OR of increased IgE* (95% CI)
Mean age months, (SD)		13.1 (1.1)	13.2 (1.1)	13.1 (1.1)	1.09 (0.97–1.24) per month
Skin colour, *n* (%)	White	916 (47)	526 (49)	390 (44)	1
	Mixed	798 (41)	442 (41)	356 (41)	0.98 (0.76–1.27)
	Black	242 (12)	111 (10)	131 (15)	0.97 (0.58–1.65)
Gender, *n* (%)	Female	939 (48)	535 (50)	404 (46)	1
	Male	1017 (52)	544 (50)	473 (54)	1.39 (0.84–2.29)
Municipality, *n* (%)	Habana Este	642 (33)	326 (30)	316 (36)	1
	Cerro	374 (19)	219 (20)	155 (18)	0.99 (0.91–1.06)
	La Lisa	282 (14)	153 (14)	129 (15)	1.59 (1.35–1.87)
	Arroyo Naranjo	658 (34)	381 (35)	277 (32)	1.11 (0.94–1.32)
Mother with paid work, *n* (%)	No	780 (40)	469 (43)	311 (35)	1
	Yes	1176 (60)	610 (57)	566 (65)	0.75 (0.55–1.02)
Any siblings, *n* (%)	No	818 (42)	454 (42)	364 (42)	1
	Yes	1138 (58)	625 (58)	513 (58)	1.18 (1.00–1.39)
Mean age of mother at birth in years (SD)	–	26.7 (6.2) *N* = 1955	26.7 (6.3)	26.7 (6.1)	0.99 (0.97–1.01) per year
Family history of asthma, *n* (%)	No	917 (47)	540 (50)	377 (43)	1
	Yes	1039 (53)	539 (50)	500 (57)	1.21 (0.88–1.67)
Reported eczema, *n* (%)	No	1284 (66)	737 (68)	547 (62)	1
	Yes	672 (34)	342 (32)	330 (38)	0.89 (0.51–1.57)
Reported wheeze, *n* (%)	No	1084 (55)	612 (57)	472 (54)	1
	Yes	872 (45)	467 (43)	405 (46)	1.03 (0.64–1.67)
Diagnosed allergic rhinitis, *n* (%)	No	677 (35)	383 (35)	294 (34)	1
	Yes	1279 (65)	696 (65)	583 (66)	1.09 (0.73–1.63)
Parasite infection, *n* (%)	No	686 (96)	98 (97)	588 (96)	1
	Yes	26† (4) *N* = 712	3 (3)	23 (4)	1.15 (0.21–6.25)

* Elevated IgE defined as above median value.

† *Endolimax lana* (six cases), *Giardia lamblia* (15 cases) and *Entamoeba histolytica* (five cases).

In the final-adjusted analysis, there were significant differences in the risk of having higher serum IgE levels for those who lived in La Lisa (odds ratio OR: 1.51: 95% confidence intervals CI: 1.28–1.79) and Arroyo Naranjo (OR 1.44; 95% CI: 1.15–1.82) compared with those who live in Habana del Este (Table2). Children who had been breastfed for 4 months or more had a higher risk of having increased IgE (OR 1.28; 95% CI: 1.02–1.61) than those who were breastfed for 3 months or shorter. Those who reported cockroaches in their home also had a higher risk of having an increased IgE than those who did not (OR 1.30; 95% CI: 1.03–1.63). The presence of a mother who worked (OR 0.73: 95% CI: 0.54–0-97 compared with one who did not), cooking with gas and electricity (OR 0.45; 95% CI: 0.32–0.62 compared to cooking with electricity alone) and the presence of pets at home at the time of birth (OR 0.83; 95% CI: 0.70–1.00) were associated with a reduced risk of having elevated IgE (Table3).

**Table 2:** Univariate analysis of exposures and risk of elevated IgE

Variable	Definition of category	Number (%) or mean (SD)	OR of increased IgE* (95% CI)
Mother used paracetamol in pregnancy (%)	No	803 (92)	1
	Yes	74 (8)	1.20 (0.94–1.52)
Mother used aspirin in pregnancy (%)	No	800 (91)	1
	Yes	77 (9)	0.65 (0.38–1.12)
Infant's mean birth weight, kg, (SD)		3.4 (0.5) *N* = 876	1 0.96 (0.65–1.42) per Kg
Infant's mean height at birth, cm, (SD)		50.4 (2.4) *N* = 876	(0.92–1.11) per cm
Infant's mean weight, kg, (SD)		10.5 (1.6) *N* = 873	1.05 (0.92–1.19) per Kg
Infant's mean height, cm, (SD)		74.7 (3.5) *N* = 872	0.98 (0.92 1.04) per cm
Caesarean birth (%)	No	516 (59)	1
	Yes	361 (41)	0.92 (0.68–1.26)
Respiratory distress at birth (%)	No	830 (95)	1
	Yes	47 (5)	2.01 (0.85–4.75)
Breastfeeding ≥4 months	No	385 (44)	1
	Yes	492 (56)	1.23 (1.01–1.50)
Insect sting allergy (%)	No	387 (44)	1
	Yes	489 (56)	1.45 (0.92–2.29)
Paracetamol use by infant including kogrip (%)	No	661 (75)	–
	Yes	216 (25)	0.98 (0.73–1.32)
State of home (%)	Good	649 (74)	1
	Regular	176 (20)	1.81 (0.77–4.27)
	Poor	52 (6)	1.23 (1.01–1.51)
No. of rooms in house (excluding bathroom and kitchen)	1	97 (11)	1
	2	242 (28)	0.70 (0.43–1.15)
	≥3	538 (61)	0.66 (0.33–1.32)
Ventilation of home (%)	Good	696 (79)	1
	Regular	125 (14)	1.66 (1.03–2.68)
	Poor	56 (6)	0.93 (0.53–1.63)
Presence of ornamental plants (%)	No	630 (72)	1
	Yes	247 (28)	0.97 (0.70–1.35)
Presence of bathroom in home (%)	No	99 (11)	1
	Yes	778 (89)	0.93 (0.63–1.38)
Mould in home (%)	No	631 (72)	1
	Yes	246 (28)	1.10 (0.61–1.97)
Child sleeps in cooking area	No	792 (90)	1
	Yes	85 (10)	1.08 (0.49–2.40)
Cook with gas (%)	No	59 (7)	1
	Yes	818 (93)	0.72 (0.52–1.00)
Cook with electricity (%)	No	688 (78)	1
	Yes	189 (22)	0.75 (0.53–1.08)
Mosquito nets in home (%)	No	320 (36)	1
	Yes	557 (64)	0.96 (0.63–1.45)
Air conditioning (%)	No	725 (83)	1
	Yes	152 (17)	0.71 (0.39–1.27)
Infant's room walls painted before birth	No	411 (47)	1
	Yes	466 (53)	0.87 (0.63–1.20)
Infant's room walls painted after birth	No	768 (88)	1
	Yes	109 (12)	0.87 (0.47–1.58)
Infant's mattress	Used	493 (56)	1
	New	384 (44)	1.06 (0.83–1.36)
Infant sleeps alone	No (not alone)	396 (45)	1
	Yes (alone)	481 (55)	1.03 (0.63–1.69)
Daily use of soap	No	27 (3)	1
	Yes	850 (97)	1.26 (0.70–2.25)
Use of shampoo	No	259 (30)	1
	Yes	618 (70)	0.96 (0.66–1.41)
No. of people in household	2	101 (12)	1
	3	226 (26)	0.63 (0.31–1.26)
	4	241 (27)	0.62 (0.25–1.54)
	5	146 (17)	0.72 (0.41–1.28)
	≥6	163 (19)	0.66 (0.27–1.62)
Eats vegetables	No	159 (18)	1
	Yes	718 (82)	0.87 (0.62–1.21)
Eats fruit	No	81 (9)	1
	Yes	796 (91)	0.97 (0.37–2.54)
Maternal smoking during pregnancy	No	815 (93)	1
	Yes	62 (7)	0.94 (0.37–2.39)
Mother currently smokes	No	710 (81)	1
	Yes	167 (19)	1.08 (0.88–1.33)
Father currently smokes	No	588 (67)	1
	Yes	289 (33)	0.97 (0.87–1.09)
Number of smokers in home	0	436 (50)	1
	1	223 (25)	1.19 (0.95–1.49)
	2	142 (16)	0.95 (0.72–1.25)
	≥3	76 (9)	0.98 (0.68–1.42)
Any grandparents smoke	No	365 (42)	1
	Yes	512 (58)	1.02 (0.74–1.41)
Pets in home at time of birth	No	561 (64)	1
	Yes	316 (36)	0.86 (0.73–1.01)
Pets in home now	No	566 (65)	1
	Yes	311 (35)	1.03 (0.80–1.34)
Rodents in home	No	749 (85)	1
	Yes	128 (15)	1.12 (0.55–2.29)
Cockroaches in home	No	633 (72)	1
	Yes	244 (28)	1.26 (0.99–1.60)
Air pollution near home	No	614 (70)	1
	Yes	263 (30)	0.97 (0.65–1.45)
Child attended day care/nursery	No	753 (86)	1
	Yes	124 (14)	0.97 (0.39–2.40)

IQR, interquartile range; OR, odds ratio.

* Elevated IgE defined as above median value.

**Table 3:** Final multivariate analysis of exposures and risk of elevated IgE

Variable	Definition of category	Number (%)	Number (%) with increased IgE	Adjusted OR for increased IgE* (95% CI)
Gender	Female	404 (46)	184 (45.5)	1
	Male	473 (54)	254 (53.7)	1.35 (0.80–2.27)
Municipality	Habana del Este	316 (36)	150 (47.5)	1
	Cerro	155 (18)	73 (47.1)	0.90 (0.80–1.02)
	La Lisa	129 (15)	76 (58.9)	1.51 (1.28–1.79)
	Arroyo Naranjo	277 (32)	139 (50.2)	1.44 (1.15–1.82)
Breastfeeding for >4 months	No	385 (44)	181 (47.0)	1
	Yes	492 (56)	257 (52.2)	1.28 (1.02–1.61)
Mother in paid employment	No	311 (35)	170 (54.7)	1
	Yes	566 (65	268 (47.4)	0.73 (0.54–0.97)
Presence of cockroaches in home	No	633 (72)	306 (48.3)	1
	Yes	244 (28)	132 (54.1)	1.30 (1.03–1.63)
Cooking source	Electricity only	59 (7)	34 (57.6)	1
	Gas only	688 (78)	354 (51.5)	1.01 (0.73–1.39)
	Gas & electricity	130 (15)	50 (38.5)	0.45 (0.32–0.62)
Pets at home at birth	No	561 (64)	288 (51.3)	1
	Yes	316 (36)	150 (47.5)	0.83 (0.70–1.00)

OR, odds ratio.

* Defined as above median value.

## Discussion

To our knowledge, this is the largest population-based study to study the association between environmental exposures and an increased serum IgE in one-year-old infants. Our data demonstrate that being breastfed for 4 months or more and living in a home with cockroaches is associated with an increased IgE, while having pets in the home at birth, a mother who worked or living in a household that uses both gas and electricity for cooking were associated with a lower serum IgE level. Together, these indicate that IgE levels are lower among children from relatively affluent families, suggesting that the environmental impact of differential socio-economic status is clearly evident at the age of 12–15 months.

The strengths of our data include the randomly selected population-based study from which these infants were recruited and the detailed phenotypic information collected on a number of environmental exposures. As the paucity of comparable studies in this year group demonstrates, this is a difficult population to obtain serum samples from. The relatively high number of infants who provided blood samples to allow measurement of total serum IgE is, hence, a major strength of our analysis, giving more power to detect significant associations with lifestyle risk factors. We were fortunate to have faeces samples from 712 infants in our data set. The fact that these demonstrated a very low prevalence of parasite infection is not surprising as infants of this age live in a very protected environment and would not be expected to be exposed to the contaminated water or soil, which may mediate parasite infection. There was no association observed between parasite infection and IgE levels, possibly as a consequence of this low prevalence of parasite infection. This is an important negative observation, as without it, we would speculate as to the role of parasite infection in driving the IgE immune response in our analysis.

There are a number of limitations that need to be borne in mind when critically evaluating these data. Firstly, the infants who gave blood samples were slightly different to those who did not in terms of skin colour, residential municipality, having a mother who had paid work and having eczema symptoms. We are unable to clarify why these groups were less likely to give a blood sample with the data collected as we did not ask specific questions on this issue. This does not invalidate the associations we observed within our data set, but may restrict the generalisability to other populations. Unfortunately, we do not have data on the umbilical cord IgE that has been shown to be strongly associated with serum IgE in 9-month infants in the only other population-based study of factors that impact on infant IgE that we are aware of (Holonen *et al*. 1991), and were thus unable to explore how the associations we have observed may be modified by serum IgE at birth, and other biological exposures that may occur *in utero*.

The study of environmental factors that modify serum IgE in young infants is an important area of research as at this stage in life the immune system is maturing and hence relatively plastic to external influences. There have been many studies demonstrating the association between increased serum IgE levels and a greater likelihood of asthma symptoms (Peat *et al*. 1996; Sherrill *et al*. 1999; Sunyer *et al*. 2000; Simpson *et al*. 2005), although few data sets with IgE measurements from the first 2 years of life (excluding birth). We are not aware of any other comparable population-based studies of serum IgE in infants aged 12–15 months that we can contrast our results with. Increased total serum IgE in early life is associated with an increased risk of persistent wheezing at age 6 years (*P* < 0.01) in a population-based cohort of 826 infants although no association was observed for cord blood (Martinez *et al*. 1995). These data from Martinez *et al*. (1995) reported a median value of serum IgE of 3.2 to 9.9 IU/ml in a population of infants aged 9 months who were living in Tucson, Arizona in 1980 to 1984, which can be compared with our median value of 35 IU/ml for infants living in Havana, Cuba, in 2010 to 2011. Obviously, there are many explanations for the wide differential in serum IgE between these two populations, with age and geographical location constituting two possibilities.

Serum IgE is an antibody that probably has evolved in humans to protect against parasite infection (Gounnl *et al*. 1994; Cooper *et al*. 2008). Although we did not see any association between higher serum IgE levels and parasite infection, the prevalence of parasite infection was low at 4% of the study population who provided stool samples. This probably reflects the young age of the infants studied, as they will not have had much exposure to soil and dirt floors and hence not yet had the chance to be infected. Data from a rural area in Cuba reported a prevalence of any helminth infection of 22% in children (mean age of 8 years) (Wordemann *et al*. 2008), and this demonstrates that exposure to parasites is common in certain regions in the country, but this is not necessarily true in our urban population. However, it is likely that in the absence of parasite infection, other environmental exposures will also increase the probability of an elevated serum IgE, and this would explain the associations observed in our data.

The association between being breastfed for 4 months or more and increased IgE was unexpected as it has not been reported previously. There have been many studies of the impact of breastfeeding on the subsequent development of allergic disease and despite the undoubted health benefits of breastfeeding on child health, the impact on allergic disease is not fully understood (Wold 2006; Revalas & Katasos 2012). One of the well-recognised benefits of breastfeeding is the transfer of maternal immunity in the form of immunoglobulin A (IgA) to the infant (Hanson & Korotkova 2002) and protection against infection (AAP 2012), and breastfed infants have lower mortality attributable to infections than those who are not breastfed (Arifeen *et al*. 2001). Breastfeeding has been reported to be associated with a lower incidence of parasitic disease (Bilenko *et al*. 2008), and we speculate that one potential mechanism to explain this is the stimulation of endogenous IgE levels that may be advantageous to infant survival, particularly from an evolutionary perspective (i.e., those individuals with higher IgE levels may be more likely to thrive and survive until adulthood, increasing the chances of having offspring).

Our finding that having a pet in the home at the time of birth is associated with lower IgE is consistent with the previous observations that pet exposure in the first year of life had a lower frequency of allergic rhinitis at 7–9 years of age and of asthma at age 12–13 years (Hesselmar *et al*. 1999). Exposure to cat allergen in the early years of life is associated with lower risk of sensitisation and asthma (Platts-Mills *et al*. 2001). Unfortunately, we were unable to collect data on the type of pet living in houses of our study population, or of cat-specific IgE sensitisation, although cats are common in Cuba.

There is no obvious plausible biological explanation why cooking with both gas and electricity (compared with just electricity) and having a mother who works are both also associated with lower IgE levels. We, thus, speculate that the latter may be a proxy measure of relatively increased affluence, as while we have tried to adjust for household income and socio-economic status in Cuba, it is likely that this is at best an imprecise measure due to undeclared earnings or money transfers from overseas. Previous data of women living in an urban area of the USA demonstrate that total serum IgE is inversely associated with levels of affluence (Lewis *et al*. 2001). Affluence may be associated with less psychological stress and better housing conditions, both factors that are associated with lower levels of allergic disease (Wright *et al*. 2002; Mendell *et al*. 2011). The association between the presence of cockroaches in the home and an increase in the infants' IgE levels could be another manifestation of lower affluence or alternatively a consequence of a higher burden of microbiological exposure stimulating the whole immune system, resulting in higher IgE levels. The cockroach is well recognised as an environmental exposure that is associated with increased levels of allergic disease in sensitized individuals in urban areas (Rosenstreich *et al*. 1997), and this may explain the association with increased IgE. Unfortunately, we do not have data on cockroach-specific IgE to clarify this issue, although recent data has reported that increased total IgE is positively correlated with allergen-specific IgE levels that included those from cockroaches (Hani 2012).

In summary, we have identified that living in a home that contains cockroaches and breastfeeding for 4 months or more are associated with higher total serum IgE, while living in a household that has pets at the time of birth, cooks with gas and electricity and having a mother that works outside the home is associated with lower serum total IgE. In probability, increased total IgE will be a consequence of a multitude of environmental exposures. As increased total serum IgE in the first year of life is associated with an increased risk of persistent wheezing at age 6 years (Martinez *et al*. 1995), understanding these relations will increase knowledge of the immune responses that may have evolved from protecting us against parasites to increasing our risk of allergic disease. In the future, we hope that this will allow manipulation of these aberrant biological responses for therapeutic benefit to design interventions available to prevent and treat allergic disease.

## Appendix

### HINASIC study group

The HINASIC study group (Historia Natural de la Sibilancia en Cuba/National History of Wheezing in Cuba) consists of the following individuals: *National Institute of Hygiene, Epidemiology and Microbiology*: Menocal-Heredia L, Caraballos-Sánchez Y, Quintana R, Rodríguez-Bertheau AM, Rosado-García FM, Carmen-Hinojosa M, Varona-Pérez P. *Hospital Universitario Pediátrico Docente Centro Habana*: Rivero R., Muñoz-Pérez J, González-Morfa C. *Municipality of Arroyo Naranjo*: Zaldívar-Ricardo D, Diburt-Amita M, Álvarez-Valdez G, Alfonso-Hernández A, Álvarez-Valdez V, Magaña-Álvarez Y, Figueroa-Barreto Z, Sardiñas-Báez N, Del Toro F, Velásquez-Pérez Y, Felpeto-Fuentes M, Gainza-Bueno Y, Esquivel-Barrios GM, Suárez-Paz M, Magaña-Álvarez BJ, Carménate-Fernández A, Hidalgo-Mederos R, Hidalgo-Mederos L, Silva D, Comas-Fonseca G, Lazaga-Cala DM, Kessel Díaz O. *Municipality of La Lisa:* Llopis-Pupo I, Rudy-Colebroork L, Loynaz-González M, Ortiz-Hernández ML, Castillo-Bu M, Betancourt-López M, Gutiérrez-Mendoza ER, Rodríguez-Trujillo N, Pozo-Herrera P, Cruz-Acosta S, Montejo-Guerra VM, Gómez-Suliman V, Vega-Enríquez Y. *Municipality of Cerro*: Pando CR, Cortina-Mena I, Díaz-Giraldino A, Marrero-Sosa M, Matos-Ramos C, Betancourt-Orue M, Torres Zulueta RM, Alba Monteagudo O, Valle-López M, Ferrer-Ceruto Y, Damas-Martínez A, Peñalver-Pérez M. *Municipality of Habana del Este:* Castillo- Martínez S, Pérez-Pérez IM, Bravo-Hernández PL, Martínez-Hernández A, Torriente-Barzaga N, Ávila-Rodríguez I, Navarro-Ruiz M, Díaz-Hernández K, Sarduy-Flores R, Sánchez-Díaz E, Zubizarreta-Seguí L, Roque-Pereira G, Corona-Carnero Y, Rafols-Turró M, Cobas-Espino T, Castillo-Hernández N, Tenreiro-Vilda GC, Pulido-Díaz VI, Oropesa-Varona MJ, Luís-Avilés R, Santos-Smith K, Serrano-González T, Vázquez-Lazo B, Pupo-Portal Tania, Torres-Martínez MC, Betancourt-Cabreras I, Cid-Morell Y, Suárez-Quiñones R, García-Pérez K, Griñán-Ramos JA, Calzado-Herrera Y, Rizo-Ramos MN, Verdecia G, García-Sotolongo MB, Del Río-Díaz A, Abreu-Quijano JF, Romeo-Ravelo F.
